# Development and validation of nomograms for predicting overall survival and cancer-specific survival in elderly patients with locally advanced gastric cancer: a population-based study

**DOI:** 10.1186/s12876-023-02749-9

**Published:** 2023-04-11

**Authors:** Yuqi Sun, Zequn Li, Yulong Tian, Chao Gao, Benjia Liang, Shougen Cao, Xiaodong Liu, Xuechao Liu, Cheng Meng, Jianfei Xu, Hao Yang, Yanbing Zhou

**Affiliations:** 1 Department of General Surgery, Affiliated Hospital of Qingdao University, Qingdao, Shandong Province China; 2 Qilu Hospital of Shandong University, Jinan, Shandong Province China; 3 Shandong Provincial Hospital, Jinan, Shandong Province China

**Keywords:** Locally advanced gastric cancer, Elderly, Survival, Nomogram

## Abstract

**Objective:**

To evaluate the multiple factors influencing the survival of elderly patients with locally advanced gastric cancer (LAGC) and develop and validate the novel nomograms for predicting the survival.

**Methods:**

The clinical features of patients treated between 2000 and 2018 were collected and collated from the Surveillance, Epidemiology, and End Results (SEER) database and three medical centres in China, and the patients were randomly divided into a training cohort (3494), internal validation cohort (1497) and external validation cohort (841). Univariate and multivariate analyses of the prognostic values were performed to identify independent prognostic factors associated with overall survival (OS) and cancer-specific survival (CSS), and two nomogram models were developed. Harrell’s concordance index (C-index) and calibration curves were employed to assess discrimination and calibration. Decision curve analysis (DCA) and receiver-operating characteristic (ROC) curves were utilized to investigate the clinical usefulness.

**Results:**

In the SEER database, the 5-year OS of the patients was 31.08%, while the 5-year CSS of the patients was 44.09%. Furthermore, in the external validation set, the 5-year OS of the patients was 49.58%, and the 5-year CSS of these patients was 53.51%. After statistical analysis, nine independent prognostic factors of OS and CSS were identified, including age, race, tumour size, differentiation, TNM stage, gastrectomy type, lymph node metastasis (LNM), lymph node ratio (LNR) and chemotherapy. The C‐index (approximately 0.7) and calibration curve (close to the optimal calibration line) indicated satisfactory discrimination and calibration of the nomogram. DCA and ROC curves showed that the developed nomogram was superior to TNM stage.

**Conclusion:**

The novel validated nomogram could accurately predict the prognosis of individual elderly patients with LAGC and guide the selection of clinical treatment measures.

## Introduction

Gastric cancer (GC) is a complex gastrointestinal malignancy that has the fifth highest incidence of any cancer type worldwide [1]. To date, radical resection is the cornerstone in the treatment of resectable gastric cancer. With the continuous progress of biochemical technology, chemotherapy including targeted drugs has been an emerging trend for practising precision medicine and improving the treatment effects of gastric cancer, but the overall survival rate is still not satisfactory [2]. By 2022, gastric cancer had become the fourth leading cause of cancer-related mortality worldwide [1, 3]. Meanwhile, as the worldwide population ages, the incidence of gastric cancer in elderly patients is increasing [4]. According to statistics, more than 60% of gastric cancer patients are aged 65 years [5]. The treatment of elderly gastric cancer patients (ELGC) consumes a large amount of social and medical resources and increases the heavy burden on families and society. However, few clinical studies have focused exclusively on ELGC, and limited evidence has been mainly derived from subgroup analyses.

ELGC patients have more comorbidities, decreased physiological reserves, and poor tumour immune responses, which eventually lead to immune escape and tumour metastasis [6]. Meanwhile, due to the high degree of malignancy and insidious onset, the majority of patients are in the mid-late stage of the disease when diagnosed. Recent data from the China Gastrointestinal Cancer Surgery Union showed that the proportion of people with locally advanced gastric cancer (LAGC) was as high as 70.8% [7]. In recent years, LAGC has evolved from a single surgical resection to multidisciplinary therapy centering on the role of surgery [8]. However, for elderly patients with LAGC, the clinicopathologic characteristics of these patients and the factors influencing prognosis have not been fully elucidated. Sufficient evidence-based medical evidence is lacking for the surgical treatment of elderly LAGC patients.

To provide optimal therapeutic strategies for this population, the assessment of factors affecting life expectancy has become of tremendous importance. To date, the American Joint Committee on Cancer (AJCC) TNM staging system has been widely used for the assessment of risk stratification and prognosis in oncology [9]. Among them, this staging system for gastric cancer has relied on a limited number of pathological variables (including tumour depth, lymph node metastasis, and distant metastasis), and assumed homogeneity within the same stage groups. In general, the health status of elderly patients with LAGC is highly complex and heterogeneous [10]. Long-term survival is affected by multiple factors, such as sex, tumour stage and pathological state, so relevant studies must combine demographic and epidemiological data [11]. In addition, compared with a single predictor, the establishment of a multivariate prediction model is more likely to increase the sensitivities and specificities of predicting prognosis at the macro level and improve the reliability of the conclusion.

Given the limitations of the AJCC TNM staging system, clinical prediction models (CPMs) have become popular among oncologists and patients as risk assessment tools [12]. On the one hand, CPMs are increasingly able to estimate individual risk based on patient and disease characteristics. On the other hand, CPMs could combine multiple predictors, including molecular, histological and clinical features, to improve the accuracy of prognostic estimates [13]. CPMs include disease occurrence models, diagnostic models and prognostic models [14]. Nomograms, as a common tool in CPMs, have been constructed successfully and proven to be effective in a variety of tumour diseases. For prognostic nomograms, researchers have often assigned corresponding values to different variables, and the total score was transformed into the occurrence probability of the outcome event. After the population was divided according to individual scores, different clinical interventions were implemented. Currently, nomograms have been used to identify high-risk patients, monitor and direct personalized therapeutics and improve the design of clinical trials. Technical guidelines for nomogram development have been published by the AJCC Precision Medicine Core to improve the validity and quality of research on accurate predictive models [12].

Recently, nomograms for predicting lymph node metastasis or prognosis have been widely used in the field of gastric cancer. However, a nomogram for predicting the survival of elderly patients with LAGC has yet to be developed and validated. This study aimed to evaluate multiple factors influencing the survival of gastric cancer patients with LAGC based on a retrospective population-based study. Novel nomograms for predicting the overall survival (OS) and cancer-specific survival (CSS) were developed and validated. On this basis, external validation of the prediction model was carried out to demonstrate its applicability in Asian populations.

### Patients and methods

#### Data sources

This study combined data from two sources. The data source of this retrospective training and internal validation cohort was from the Surveillance, Epidemiology, and End Results Program (SEER). At present, the SEER database consists of cancer registries from 21 geographic areas, covering approximately one-third of the American population [15]. As the largest publicly authoritative data system, the SEER database includes more than 100 sociodemographic and clinical characteristics. Moreover, the SEER data are available to the public for research purposes, and no ethics committee approval or consent procedures are needed.

In addition, data from the Affiliated Hospital of Qingdao University, Qilu Hospital of Shandong University and Shandong Provincial Hospital were used to externally validate the model. According to the prespecified protocol, all medical records were retrieved, and data were extracted by two reviewers to improve the validity. The interrater reliability between the evaluators was found to be excellent (Cohen κ index 0.9). Furthermore, we adhered to the Strengthening the Reporting of Observational Studies in Epidemiology (STROBE) guidelines for cohort studies to ensure the quality of the research [16]. All procedures were approved by the Ethics Committee of the three medical centres. Oral informed consent was obtained from all patients.

### Study population

In this study, the clinical features of 123,964 patients with stomach cancer were downloaded between 2000 and 2018 from the SEER database using SEER*Stat software (v8.3.6).

The eligibility criteria were as follows: (1) At or over the age of 65; (2) All patients had been pathologically confirmed to have gastric adenocarcinoma by preoperative gastroscopy biopsy or postoperative pathology; (3) All patients underwent radical (R0) surgical treatment consisting of gastrectomy and lymphadenectomy; (4) Histologically proven locally advanced gastric cancer patients (T1-2N + M0 or T3-4NanyM0); and (5) All the patients had complete follow-up data. The exclusion criteria were as follows: (1) Patients under the age of 65 or early gastric cancer (EGC); (2) Patients with multiple tumours, or distant metastasis; (3) Patients with confirmed pathology diagnosis of nonadenocarcinoma, GIST or a neuroendocrine tumour; (4) Patients who did not undergo gastrectomy or underwent partial gastrectomy; (5) All-cause mortality within 30 days of surgery; and (6) Patients with incomplete clinical data (medical records or follow-up data).

**Fig. 1:**
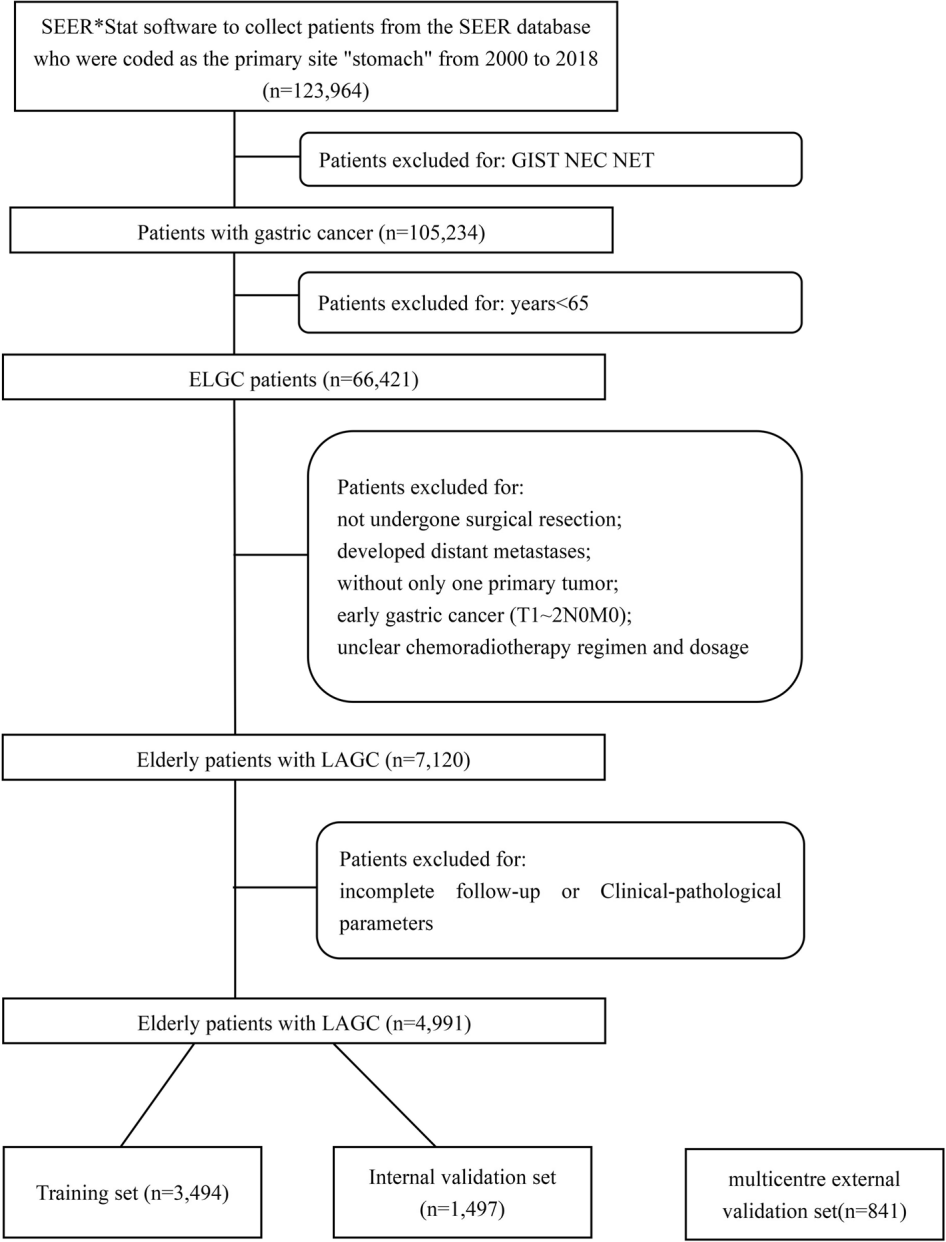
Flow chart of patient selection

The data screening process is shown in the flow diagram (Fig. 1). A total of 4991 eligible patients were included in this study. The elderly patients with LAGC were randomly divided into a training cohort (*n* = 3494) and an internal validation cohort (*n *= 1497) with an allocation of 7:3 ratio by R software. In the external validation set, 841 elderly patients with LAGC at the three medical centres were retrospectively collected and reviewed between January 2015 and December 2018.

### Observation indicators and endpoints

The main observation indices of this study included the demographics of the patients (sex, age, race, marital status at diagnosis), the clinicopathological features of the cancer (tumour location, size, tumour differentiation, histology, gastrectomy type, depth of invasion, lymph node metastasis, distant metastasis, tumour stage, chemotherapy record) and survival data (survival time and death reason). According to the specific circumstances and goals of the study, as well as the nature of the data and the relationship between the variables, this study converted the continuous variables into categorical variables in the regression analysis. It should be noted that categorizing continuous variables can be helpful in cases where there is a nonlinear relationship between the predictor and outcome variables, and it may be difficult to find a suitable model to fit. While splines can be used as an alternative, they can be computationally intensive. X-tile is a bioinformatics tool for risk factor assessment assessment and outcome-based cut-point optimization. As an alternative, the optimal cut-off values of age, tumour size and lymph node ratio (LNR) were determined using the X-tile program (X-tile software version 3.6.1, Yale University) [17], and the continuous variables were converted into classification variables.

Age was categorized into three groups: 65 ~ 70 years old, 71 ~ 80 years old, and ≥ 81 years old. Race was divided into four groups: white, black, Asian or Pacific Islander and Indian or unknown. The two marriage categories were married and unmarried (including single, widowed, divorced and informal union). Tumour size was divided into three groups (< 3.5, 3.5 ≤ tumour size < 9.5, and ≥ 9.5). The location of the tumour was divided into the cardia/fundus, body and antrum/pylorus. Tumour differentiation was defined according to the cellular differentiation degree, which may be classified as I-II and III-IV [18]. Pathology type was classified as adenocarcinoma and signet-ring cell carcinoma. The type of surgery included proximal gastrectomy, distal gastrectomy and total gastrectomy. Cancer stage was categorized according to the Staging Manual of the AJCC [9]. The positive rate of lymph node metastasis was classified into 2 groups with a cut-off of 33%. In addition, cause-specific survival (CSS) and overall survival (OS) were used as the main study endpoints. In this study, CSS was defined as the time from gastric cancer diagnosis until gastric cancer-related death or end of follow-up. OS was defined as the time to death from any cause or the end of follow-up.

### Development and validation of the nomogram

Univariate and multivariate analyses of the prognostic values were performed using the Cox proportional hazards regression model, which was fundamental to the survival prediction model. Factors with *P* < 0.10 in the univariate analyses were entered into the multivariate regression model. The covariates included in the nomogram models were selected based on the independent risk factors affecting survival. Thereafter, nomograms predicting 1-, 3- and 5-year OS as well as 1-, 3- and 5-year CSS were constructed using the “rms” package (6.2–0) of R software 3.5.0.

The goal of a forecasting model is to predict the outcome as quickly and accurately as possible. The predictive power of the nomogram was assessed by both discrimination and calibration [19]. Discrimination referred to the ability to separate patients with different outcomes and used the Harrell’s concordance index (C-index) as the measurement tool [20, 21]. Moreover, the C-index and 95% confidence interval (CI) were calculated on the basis of bootstrap resampling with 1000 replicates. A C-index of 1 indicated perfect discrimination, and a C-index of 0.5 indicated that the model was not better than random chance. The calibration of the models could be assessed using a calibration chart, which was used to evaluate the difference between the predicted probability and the actual result, and the 45-degree line denoted the optimal prediction [20]. To avoid overfitting, fivefold cross-validation was adopted for the nomogram model. Finally, the clinical usefulness of the nomogram was the last component in evaluating the value of the nomogram. Decision curve analysis (DCA) was utilized to investigate whether the nomogram-assisted decisions effectively improved the outcome for individual patients [22].

### Statistical analysis

The randomization sequences were generated using the RANDBETWEEN function in Microsoft Excel. The difference distribution of the categorical variables between the subgroups was assessed using Pearson’s χ^2^ test and Fisher’s exact test. OS and CSS curves were plotted using the Kaplan–Meier method, and the differences between the survival curves were evaluated with the log-rank test. Receiver operating characteristic (ROC) curves were plotted by SPSS, and the area under the ROC curve (AUC) was determined to evaluate the accuracy of the model. All statistical graphics and analyses were performed using SPSS software (IBM SPSS Statistics 26.0) or R software (Version 3.5.0). *P* values < 0.05 were regarded to indicate statistically significant differences.

## Results

### Baseline clinical characteristics of the patients

The patient demographics and clinicopathological data for the three cohorts are shown in Table 1, including the training cohort (*n* = 3494), internal validation cohort (*n* = 1497), and external validation cohort (*n* = 841). We observed that the proportions of male patients were higher than those of female patients among the elderly patients with LAGC. This difference seemed to be more pronounced in the Asian populations. This was supported by a recent study that found that the incidence rate of gastric cancer was 1.8 to 2.0 times higher among men than women [23]. Furthermore, approximately half of the gastric cancer patients were 71–80 years old. Advanced age was associated with functional impairment, comorbidities and frailty, all of which increase the risk of radical surgical resection [8, 24]. The special physical state of the elderly and stress response after surgery also caused only 50% of the LAGC patients to receive adjuvant chemoradiotherapy. In the SEER database, more than 70% of the patients were white or black, with differences in the demographic distribution. To further illustrate the general applicability of the prediction model for LAGC in elderly individuals, we used external data to validate the model against Asian populations.

**Table 1:** Patient and tumour characteristics in the training and validation cohorts

Characteristic	Training Cohort (*n* = 3494)	Internal validation Cohort (*n* = 1497)	*P*-value^a^	External validation Cohort (n = 841)	*P*-value^b^
	Mean ± SD /No (%)	Mean ± SD /No (%)		Mean ± SD /No (%)	
Sex			0.472		< 0.001^*^
Male	2025(58.0)	884(59.1)		633(75.3)	
Female	1469(42.0)	613(40.9)		208(24.7)	
Age (years)			0.620		< 0.001^*^
65 ~ 70	931(26.6)	392(26.2)		319(37.9)	
71 ~ 80	1606(46.0)	710(47.4)		435(51.7)	
≥ 81	957(27.4)	395(26.4)		87(10.3)	
Race			0.508		< 0.001^*^
White	2225(63.7)	962(64.3)		0	
Black	443(12.7)	170(11.4)		0	
Asian or Pacific Islander	799(22.9)	350(23.4)		841(100.0)	
Indian or unknown	27(0.8)	15(1.0)		0	
Marital status			0.365		0.102
Married	2009(57.5)	840(56.1)		510(60.6)	
Unmarried	1485(42.5)	657(43.9)		331(39.4)	
Tumor size (cm)			0.404		0.001^*^
< 3.5	829(23.7)	367(24.5)		164(19.5)	
3.5 ≤ Tumor size < 9.5	2249(64.4)	971(64.9)		603(71.7)	
≥ 9.5	416(11.9)	159(10.6)		74(8.8)	
Tumor location			0.579		0.001^*^
Cardia/ Fundus	967(27.7)	426(28.5)		201(23.9)	
Body	931(26.6)	378(25.3)		187(22.2)	
Antrum/ Pylorus	1596(45.7)	693(46.3)		453(53.9)	
Tumor differentiation			0.244		< 0.001^*^
I-II	1047(30.0)	424(28.3)		154(18.3)	
III-IV	2447(70.0)	1073(71.7)		687(81.7)	
Histology			0.762		0.583
Adenocarcinoma	2918(83.5)	1245(83.2)		713(84.8)	
Signet-ring cell carcinoma	576(16.5)	252(16.8)		128(15.2)	
Gastrectomy type			0.641		< 0.001^*^
Distal gastrectomy	1696(48.5)	716(47.8)		491(58.3)	
Proximal gastrectomy	175(5.0)	68(4.5)		130(15.5)	
Total gastrectomy	1623(46.5)	713(47.6)		220(26.2)	
Depth of invasion			0.840		< 0.001^*^
T1	226(6.5)	105(7.0)		23(2.7)	
T2	316(9.0)	132(8.8)		59(7.0)	
T3	1709(48.9)	718(48.0)		387(46.0)	
T4	1243(35.6)	542(36.2)		372(44.2)	
Number of positive LN			0.755		0.015^*^
N0	770(22.0)	304(20.3)		206(24.5)	
N1	916(26.2)	398(26.6)		209(24.9)	
N2	813(23.3)	356(23.8)		229(27.2)	
N3a	694(19.9)	307(20.5)		148(17.6)	
N3b	301(8.6)	132(8.8)		49(5.8)	
AJCC TNM stage^#^			0.172		0.001^*^
I	150(4.3)	70(4.7)		13(1.5)	
II	1438(41.2)	574(38.3)		355(42.2)	
III	1906(54.5)	853(57.0)		473(56.2)	
Lymph node metastasis			0.173		0.062
No	770(22.0)	304(20.3)		206(24.5)	
Yes	2724(78.0)	1193(79.7)		635(75.5)	
Lymph node positive rate			0.965		0.032^*^
≤ 0.33	2152(61.6)	923(61.7)		558(66.3)	
> 0.33	1342(38.4)	574(38.3)		283(33.7)	
Postoperative chemoradiotherapy			0.061		0.011^*^
No	2162(61.9)	884(59.1)		477(56.7)	
Yes	1332(38.1)	613(40.9)		364(43.3)	

^#^ The 8th AJCC classification criteria; LN: lymph node; *P < 0.05; a means comparing the training cohort with the internal validation group; b means comparing the SEER database with the external validation group

In addition, due to the absence of clinical manifestation in early disease, more than 80% of the patients presented with a tumour size larger than 3.5 cm. Nearly half of the patients had tumours that were located in the gastric antrum or pylorus. For tumours located in the lower or middle third of the stomach, distal gastrectomy combined with gastric lymph node resection was the procedure of choice. All patients underwent R0 resection. It is still noteworthy that poorly differentiated tumours accounted for 70%-81%, implying that LAGC has highly malignant behaviour. Moreover, over 75% of the patients were positive for lymph node metastasis in all three cohorts.

### Survival analysis for elderly patients with LAGC

**Fig. 2:**
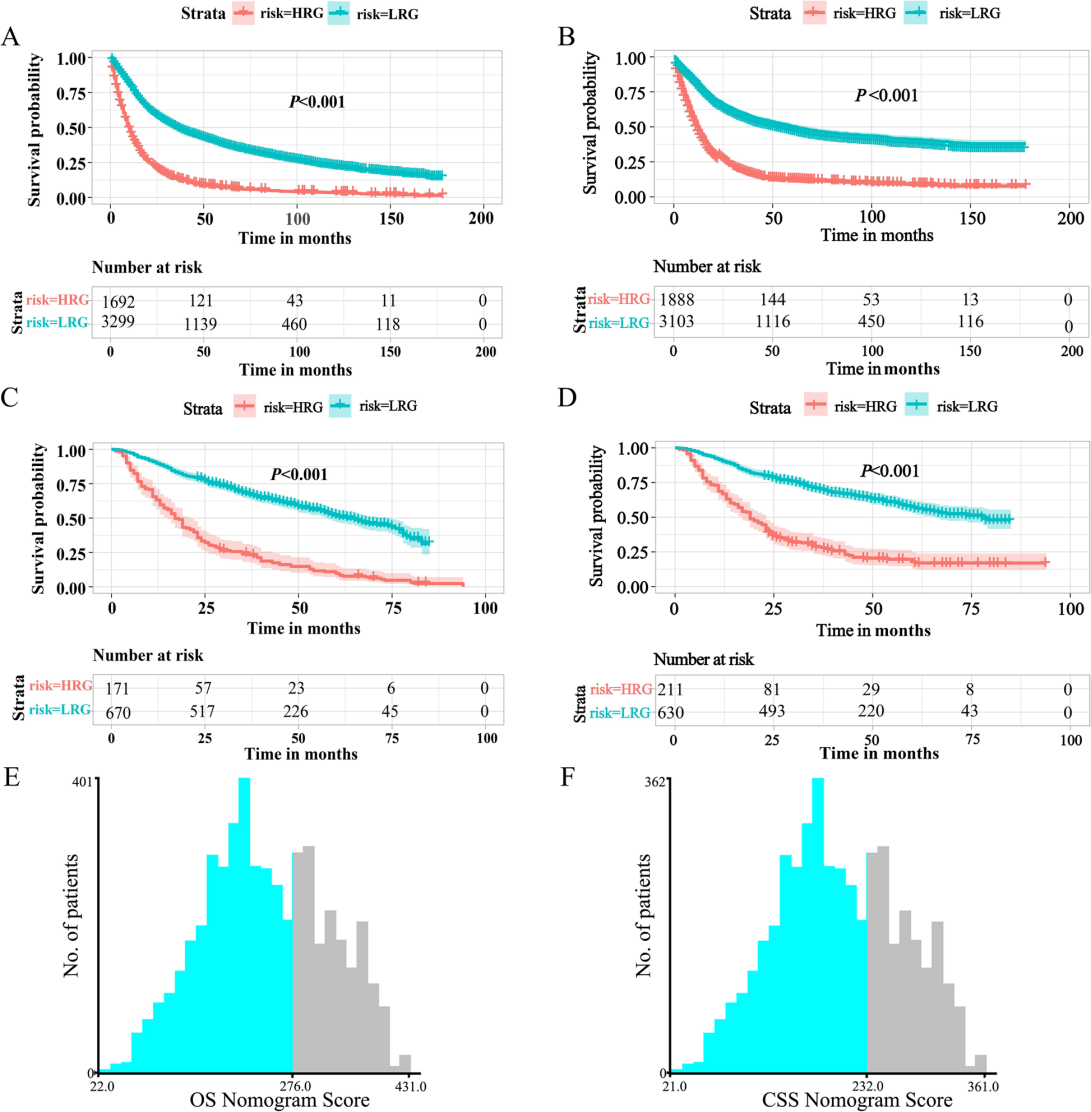
Kaplan-Meier survival curves for the high-risk and low-risk groups based on risk scores. OS and CSS curves in the SEER database (**A**, **C**) and in the three medical centres (**B**, **D**). The optimal cut-off values for the OS (**E**) and CSS (**F**). Abbreviations: *OS* overall survival, *CSS* cancer-specific survival

In the SEER database, the overall follow-up period was from 1 to 178 months in the elderly patients with LAGC, and the median follow-up period covered 19 months. During the follow-up period, a total of 1,141 patients were alive, and 3,850 of the GC patients died, including 2,951 cancer-related deaths. In the training cohort, the 5-year OS of the patients was 30.65%, while the 5-year CSS of the patients was 44.09%. The 5-year OS and CSS rates for the internal validation were 32.06% and 44.09%, respectively. Furthermore, in the external validation set, the median follow-up period was 34 (range 1–94). Of 841 elderly patients with LAGC, 424 patients died, 391 for cancer-related reasons. The 5-year OS of these patients was 49.58%, and the 5-year CSS of these patients was 53.51% (Fig. 2).

In the present study, as shown in Tables 2 and 3, the characteristics of the patients and tumour-related and treatment-related factors were included in the univariate Cox regression analysis. The univariate analysis revealed that age at presentation, race, marital status, tumour size, location, differentiation, T stage, N stage, TNM stage, gastrectomy type, LNM, LNR and chemotherapy were significantly associated with both OS and CSS. Subsequently, the above factors were included in a multivariate Cox regression model. Regarding OS, multivariate analyses showed that age (*P* < 0.001), race (*P* < 0.001), tumour size (*P* < 0.001), differentiation (*P* = 0.004), TNM stage (*P* < 0.001), gastrectomy type (*P* < 0.001), LNM (*P* = 0.007), LNR (*P* < 0.001) and chemotherapy (*P* < 0.001) were the independent risk factors identified for OS. In terms of CSS, age (*P* < 0.001), race (*P* = 0.002), tumour size (*P* < 0.001), differentiation (*P* < 0.001), TNM stage (*P* < 0.001), gastrectomy type (*P* < 0.001), LNM (*P* = 0.007), LNR (*P* < 0.001) and chemotherapy (*P* < 0.001) remained independent risk factors for CSS.

**Table 2:** Univariate and multivariate Cox proportional hazard analyses of OS in the training cohort

Variable	Univariate analysis			Multivariate analysis		
	β-coefficient	HR (95%CI)	*P-*value	β-coefficient	HR (95%CI)	*P*-value
Sex	-0.024	0.977(0.905–1.054)	0.542			
Age (years)			< 0.001^*^			< 0.001^*^
65 ~ 70		Reference			Reference	
71 ~ 80	0.274	1.315(1.915–1.446)	< 0.001^*^	0.276	1.318(1.197–1.452)	< 0.001^*^
≥ 81	0.511	1.666(1.503–1.848)	< 0.001^*^	0.431	1.539(1.381–1.716)	< 0.001^*^
Race			< 0.001^*^			< 0.001^*^
White		Reference			Reference	
Black	-0.005	0.995(0.886–1.117)	0.928	0.096	1.101(0.979–1.238)	0.107
Asian or Pacific Islander	-0.209	0.812(0.739–0.892)	< 0.001^*^	-0.173	0.841(0.765–0.925)	< 0.001^*^
Indian or unknown	0.042	1.043(0.678–1.604)	0.848	0.220	1.246 (0.808–1.923)	0.319
Marital status	0.161	1.175(1.089–1.267)	< 0.001^*^			
Tumor size (cm)			< 0.001^*^			< 0.001^*^
< 3.5		Reference			Reference	
3.5 ≤ Tumor size < 9.5	0.227	1.255(1.144–1.378)	< 0.001^*^	0.071	1.074(0.976–1.182)	0.144
≥ 9.5	0.601	1.824(1.598–2.082)	< 0.001^*^	0.312	1.366(1.192–1.576)	< 0.001^*^
Tumor location			0.003^*^			
Cardia/ Fundus		Reference				
Body	-0.031	0.969(0.876–1.072)	0.543			
Antrum/ Pylorus	-0.144	0.866(0.792–0.947)	0.002^*^			
Tumor differentiation	0.274	1.316(1.210–1.431)	< 0.001^*^	0.127	1.135(1.041–1.238)	0.004^*^
Gastrectomy type			< 0.001^*^			< 0.001^*^
Distal gastrectomy		Reference			Reference	
Proximal gastrectomy	0.031	1.031(0.866–1.229)	0.729	0.015	1.015(0.851–1.211)	0.866
Total gastrectomy	0.214	1.238(1.146–1.338)	< 0.001^*^	0.165	1.180(1.090–1.277)	< 0.001^*^
Histology	0.201	1.223(1.108–1.352)	< 0.001^*^			
Depth of invasion			< 0.001^*^			
T1		Reference				
T2	0.227	1.254(1.003–1.568)	0.047^*^			
T3	0.608	1.836(1.528–1.208)	< 0.001^*^			
T4	0.957	2.604(2.161–3.138)	< 0.001^*^			
Number of positive LN			< 0.001^*^			
N0		Reference				
N1	0.197	1.218(1.086–1.366)	0.001^*^			
N2	0.464	1.590(1.416–1.785)	< 0.001^*^			
N3a	0.854	2.349(2.087–2.644)	< 0.001^*^			
N3b	1.031	2.803(2.414–3.255)	< 0.001^*^			
AJCC TNM stage			< 0.001^*^			< 0.001^*^
I		Reference			Reference	
II	0.297	1.345(1.076–1.681)	0.009^*^	0.271	1.312(1.039–1.656)	0.023^*^
III	1.020	2.772(2.225–3.453)	< 0.001^*^	0.754	2.125(1.685–2.680)	< 0.001^*^
Lymph node metastasis	0.505	1.657(1.506–1.825)	< 0.001^*^	0.167	1.182(1.046–1.335)	0.007^*^
Lymph node positive rate	0.793	2.209(2.045–2.387)	< 0.001^*^	0.499	1.648(1.501–1.808)	< 0.001^*^
Postoperative chemoradiotherapy	-0.410	0.664(0.613–0.718)	< 0.001^*^	-0.508	0.601(0.553–0.654)	< 0.001^*^

*HR* Hazard ratio, *CI* Confidence interval, *LN* lymph nodes, ^*^P < 0.05

**Table 3:** Univariate and multivariate Cox proportional hazard analyses of CSS in the training cohort

Variable	Univariate analysis			Multivariate analysis		
	β-coefficient	HR (95%CI)	*P-*value	β-coefficient	HR (95%CI)	*P*-value
Sex	-0.011	0.989(0.907–1.080)	0.810			
Age (years)			< 0.001^*^			< 0.001^*^
65 ~ 70		Reference			Reference	
71 ~ 80	0.243	1.275(1.144–1.420)	< 0.001^*^	0.269	1.308(1.173–1.459)	< 0.001^*^
≥ 81	0.373	1.452(1.290–1.636)	< 0.001^*^	0.326	1.386(1.223–1.569)	< 0.001^*^
Race			0.005^*^			0.002^*^
White		Reference			Reference	
Black	-0.004	0.996(0.872–1.137)	0.949	0.104	1.109(0.970–1.268)	0.129
Asian or Pacific Islander	-0.194	0.823(0.739–0.917)	< 0.001^*^	-0.167	0.847(0.759–0.944)	0.003^*^
Indian or unknown	-0.002	0.998(0.600–1.660)	0.993	0.244	1.276 (0.764–2.129)	0.351
Marital status	0.112	1.119(1.025–1.220)	0.012^*^			
Tumor size (cm)			< 0.001^*^			< 0.001^*^
< 3.5		Reference			Reference	
3.5 ≤ Tumor size < 9.5	0.265	1.303(1.196–1.452)	< 0.001^*^	0.060	1.061(0.950–1.186)	0.293
≥ 9.5	0.669	1.953(1.680–2.269)	< 0.001^*^	0.297	1.346(1.152–1.572)	< 0.001^*^
Tumor location			0.008^*^			
Cardia/ Fundus		Reference				
Body	-0.002	0.998(0.889–1.119)	0.968			
Antrum/ Pylorus	-0.138	0.871(0.786–0.966)	0.009^*^			
Tumor differentiation	0.387	1.472(1.334–1.625)	< 0.001^*^	0.188	1.207(1.089–1.337)	< 0.001^*^
Gastrectomy type			< 0.001^*^			< 0.001^*^
Distal gastrectomy		Reference			Reference	
Proximal gastrectomy	0.113	1.119(0.918–1.365)	0.266	0.094	1.099(0.900–1.341)	0.355
Total gastrectomy	0.262	1.299(1.189–1.419)	< 0.001^*^	0.192	1.212(1.106–1.327)	< 0.001^*^
Histology	0.253	1.287(1.151–1.440)	< 0.001^*^			
Depth of invasion			< 0.001^*^			
T1		Reference				
T2	0.324	1.383(1.041–1.839)	0.026^*^			
T3	0.799	1.224(1.753–2.821)	< 0.001^*^			
T4	1.259	3.520(2.772–4.471)	< 0.001^*^			
Number of positive LN			< 0.001^*^			
N0		Reference				
N1	0.303	1.353(1.176–1.558)	< 0.001^*^			
N2	0.644	1.904(1.656–2.188)	< 0.001^*^			
N3a	1.081	2.949(2.564–3.390)	< 0.001^*^			
N3b	1.311	3.709(3.137–4.386)	< 0.001^*^			
AJCC TNM stage			< 0.001^*^			< 0.001^*^
I		Reference			Reference	
II	0.402	1.494(1.118–1.998)	0.007^*^	0.397	1.488(1.101–2.011)	0.010^*^
III	1.331	3.784(2.844–5.033)	< 0.001^*^	1.006	2.735(2.030–3.685)	< 0.001^*^
Lymph node metastasis	0.697	2.008(1.784–2.260)	< 0.001^*^	0.206	1.229(1.058–1.427)	0.007^*^
Lymph node positive rate	0.957	2.604(2.385–2.843)	< 0.001^*^	0.572	1.772(1.595–1.968)	< 0.001^*^
Postoperative chemoradiotherapy	-0.350	0.705(0.644–0.771)	< 0.001^*^	-0.509	0.601(0.546–0.661)	< 0.001^*^

HR, hazard ratio; CI, confidence interval; LN, lymph nodes; ^*^P < 0.05

### Nomogram construction and validation

**Fig. 3:**
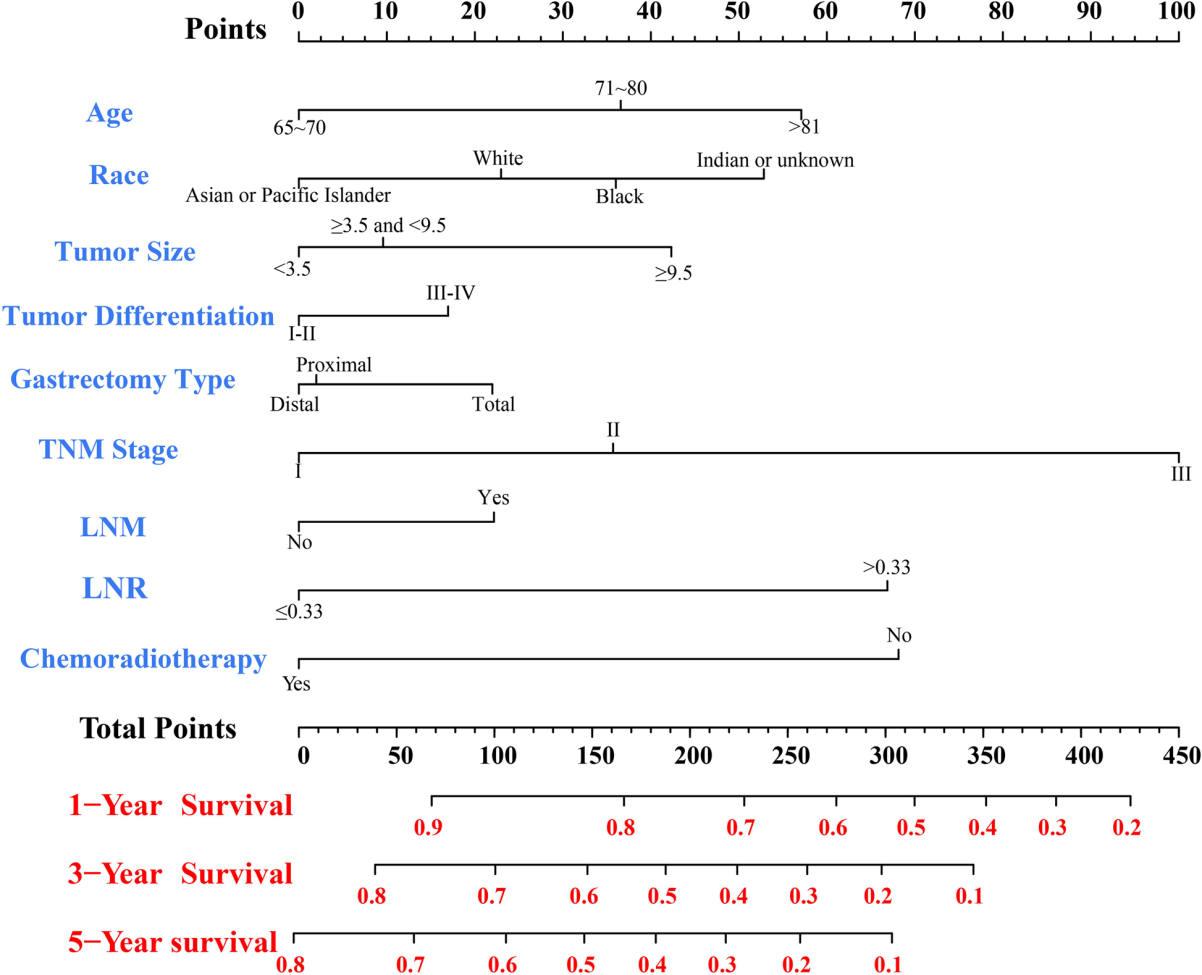
Nomogram for predicting the 1-, 3- and 5-year OS rates of elderly patients with LAGC. Higher scores indicate lower expected survival of patients. Abbreviations: *LNM* Lymph node metastasis, *LNR* Lymph node ratio, *LAGC* Locally advanced gastric cancer, *OS* Overall survival

**Fig. 4:**
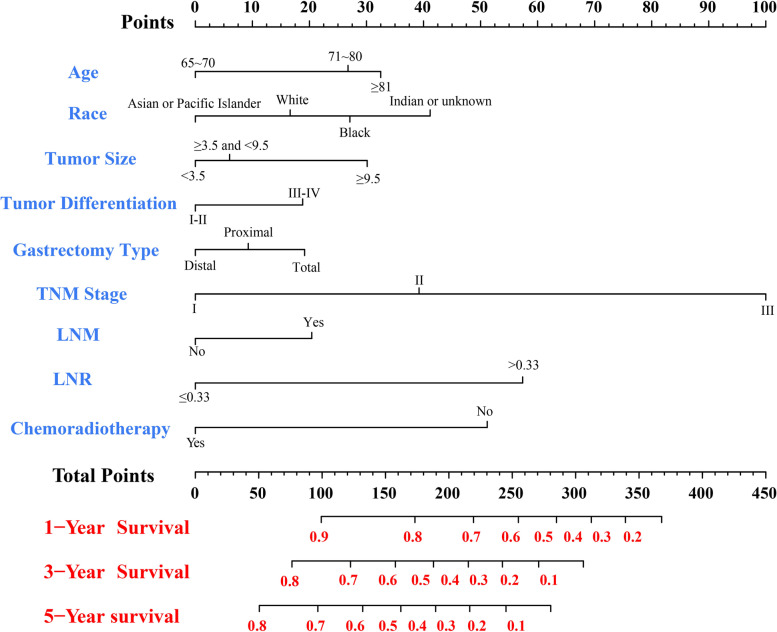
Nomogram for the prediction of 1-, 3- and 5-year CSS rates of elderly patients with LAGC. Higher scores indicate lower expected survival of patients. Abbreviations: *LNM* Lymph node metastasis, *LNR* Lymph node ratio, *LAGC* Locally advanced gastric cancer, *CSS* Cancer-specific survival

Based on the independent prognostic analysis, nomograms were constructed to evaluate the predictive ability of the 1-year, 3-year and 5-year OS and CSS in elderly patients undergoing resection for LAGC (Figs. 3 and 4). Essentially, nomograms are an excellent visualization tool to quantify the results of Cox regression equations [25]. The top of the nomogram has a reference line that rated scales to each predictor from 0 to 100. As shown in Table 4, each factor was given different values according to the regression coefficient. A greater value indicated poorer patient survival and a higher mortality risk. The total score was obtained by summing the scores of each relevant factor, and the corresponding score could be found on the “Total Points” axis. Subsequently, a vertical line was drawn down directly to the survival probability axis, and the intersection of two curves represented the probabilities of 1-, 3- and 5-year OS and CSS. In addition, the X-axis of each variable in the nomogram starts at zero to make it easier to understand and interpret the relationship between the variables based on what best serves the goals of the nomogram and the needs of the target audience.

**Table 4:** Scores of prognostic factors in the OS and CSS nomograms

Characteristic	OS nomogram	CSS nomogram
Age (years)		
65 ~ 70	0	0
71 ~ 80	37	26
≥ 81	57	32
Race		
White	23	16
Black	36	27
Asian or Pacific Islander	0	0
Indian or unknown	53	41
Tumor size (cm)		
< 3.5	0	0
3.5 ≤ Tumor size < 9.5	10	6
≥ 9.5	42	29
Tumor differentiation		
I-II	0	0
III-IV	17	18
Gastrectomy type		
Distal gastrectomy	0	0
Proximal gastrectomy	2	8
Total gastrectomy	22	19
AJCC TNM stage		
I	0	0
II	36	38
III	100	100
Lymph node metastasis		
No	0	0
Yes	22	21
Lymph node positive rate		
≤ 0.33	0	0
> 0.33	67	56
Postoperative chemoradiotherapy		
No	68	51
Yes	0	0

For nomogram validation, the discrimination and calibration of this novel model were assessed. In the training group, the c-indexes of the nomogram for the prediction of OS and CSS were 0.679 and 0.694, respectively. In the internal validation set, the c-indexes for OS and CSS prediction were 0.687 and 0.696, respectively. The c-indexes of the external validation cohort for OS and CSS all exceeded 0.7 (Table 5). From these results, it was clear that the nomograms for CSS and OS showed relatively precise discrimination ability. It is worth noting that the c-indexes of the internal and external validation cohorts were larger than those of the training cohort. The possible reason was that the difference could have been caused by the differences in demographics and survival. The prognostic variables more closely correlated with patient survival. In the future, a prospective and multicenter study will be necessary to further validate the results.

**Table 5:** C-index of the two nomogram models

Cohort	OS nomogram		CSS nomogram	
	C-index	95% CI	C-index	95% CI
Training set	0.679	0.669–0.689	0.694	0.682–0.706
Internal validation set	0.687	0.671–0.703	0.696	0.678–0.714
External validation set	0.713	0.689–0.737	0.706	0.681–0.731

**Fig. 5:**
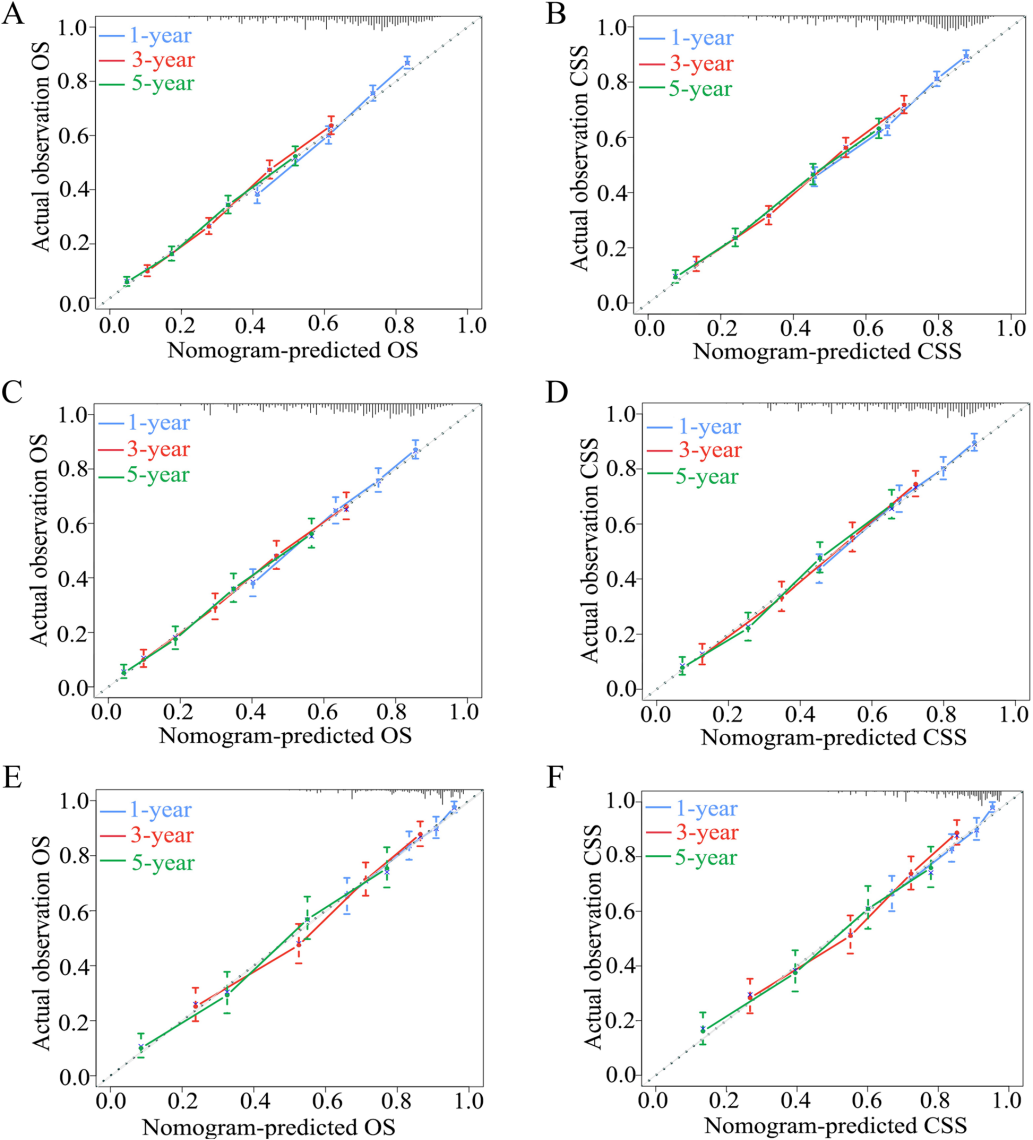
The calibration curves of the prognostic nomogram for 1-, 3-, and 5-year OS and CSS in the training set (**A**, **B**), internal validation set (**C**, **D**) and external validation set (**E**, **F**). Abbreviations: OS overall survival, CSS cancer-specific survival

In addition, as shown in Fig. 5, a calibration curve was used to calibrate the prediction model. The calibration line of all three cohorts was close to the optimal calibration line, suggesting that the model had good predictive accuracy in predicting the survival of patients for certain years.

### Evaluation quality and clinical usefulness of nomograms

**Fig. 6:**
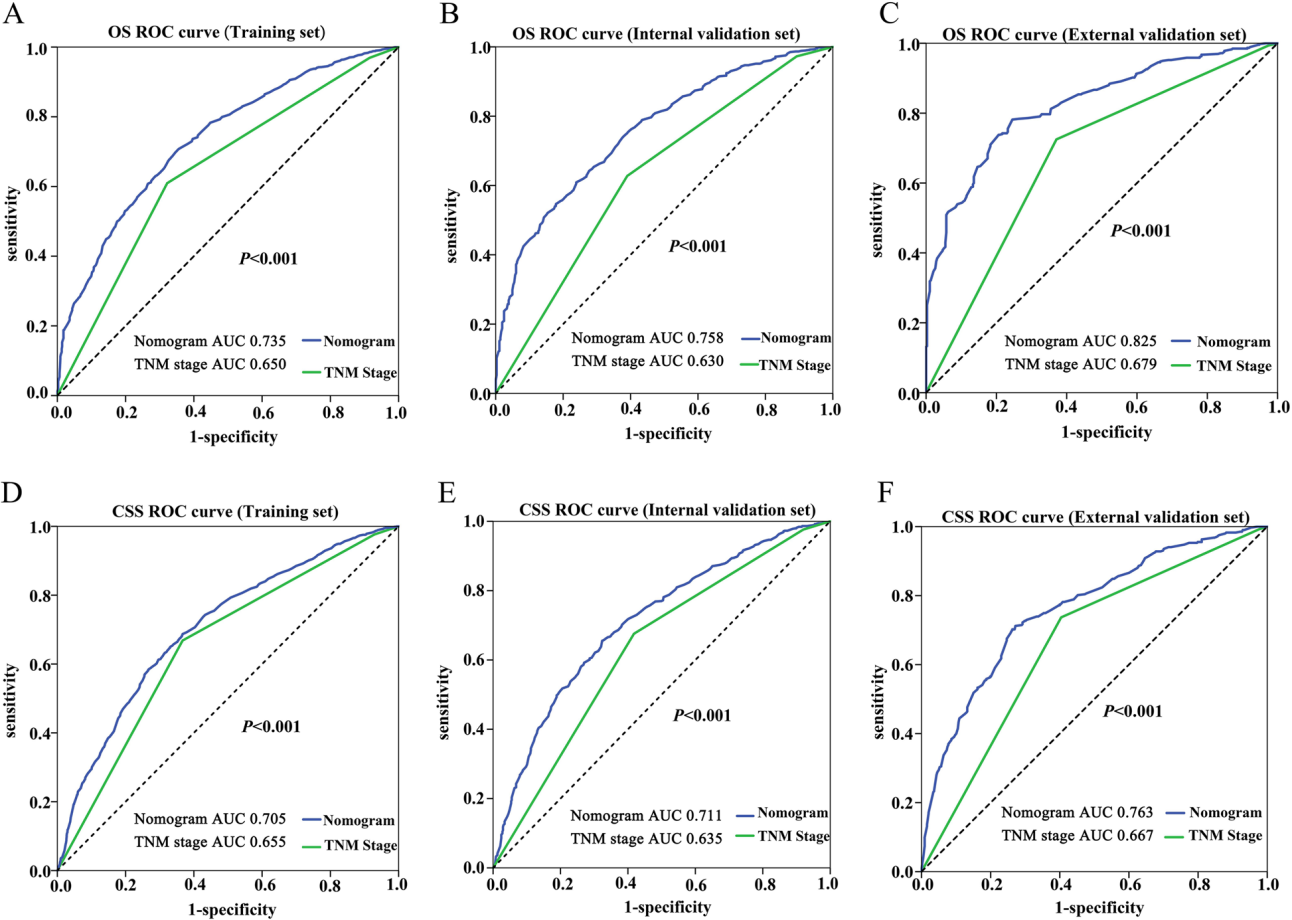
Comparison of the ROC curves between the nomogram prediction models and the 8th edition AJCC TNM staging for the prediction of OS (**A**-**C**) and CSS (**D**-**F**). Abbreviations: *OS* overall survival, *CSS* cancer-specific survival

**Fig. 7:**
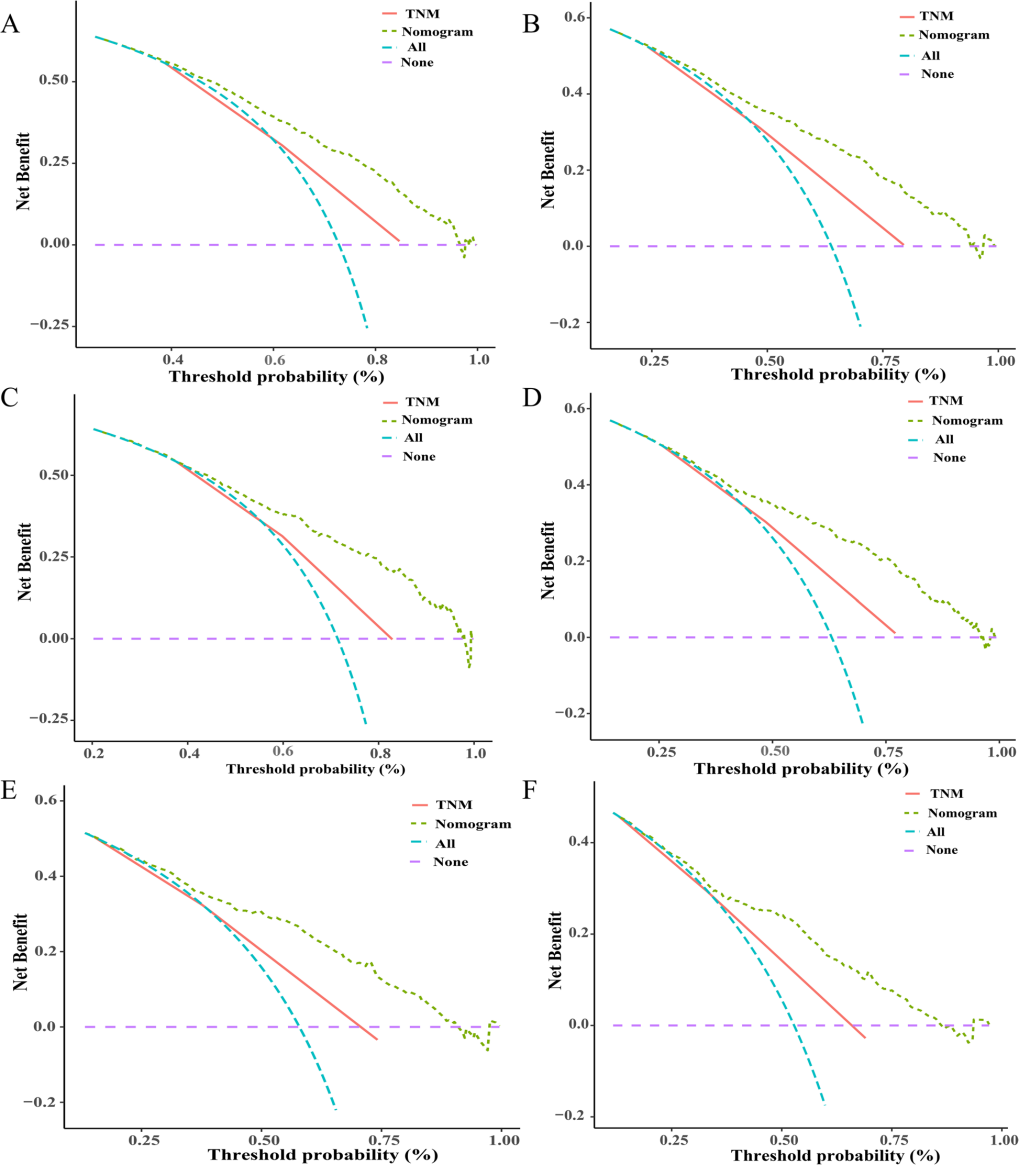
DCA of the OS nomogram models and TNM staging system in the elderly patients with LAGC in the training set (**A**), internal validation set (**C**) and external validation set (**E**). DCA of the CSS nomogram models and TNM staging system in the elderly patients with LAGC in the training set (**B**), internal validation set (**D**) and external validation set (**F**). Abbreviations: *DCA* Decision curve analysis, *LAGC* Locally advanced gastric cancer, *OS* Overall survival, *CSS* Cancer-specific survival

The last component of the nomogram performance assessment was clinical usefulness. To evaluate the prognostic value of the nomogram and TNM staging in elderly patients with LAGC, ROC curves were established, and the AUC was assessed (Fig. 6). The AUCs in the nomogram for OS and CSS prediction were 0.735 (95% CI, 0.716 to 0.755) and 0.705 (0.688–0.723), respectively, and the AUCs of TNM staging were 0.650 (0.628–0.672) and 0.655 (0.636–0.674), respectively, in the training cohort. In the internal validation set, the AUCs of the nomogram for OS and CSS prediction were 0.758 (0.732–0.785) and 0.711 (0.684–0.737), respectively. The AUCs of the TNM staging were 0.630 (0.597–0.664) and 0.635 (0.606–0.664). Moreover, the AUCs of the generated nomograms for OS and CSS were 0.825 (0.798–0.853) and 0.763 (0.731–0.795), respectively, which were significantly larger than those of TNM staging (0.679 (0.642–0.715) and 0.667 (0.630–0.704), *P* < 0.001). It was considered that the predictive efficacy of the nomogram was better than that of TNM staging. In addition, decision curve analysis showed that the established nomogram displayed good net clinical benefit compared with the traditional TNM staging system (Fig. 7).

## Discussion

Worldwide, gastric cancer is one of the most malignant neoplasms and has a high mortality rate. In recent years, with the acceleration of the ageing population and longer life expectancy, the incidence and absolute number of cases of gastric cancer in the elderly has been increasing, and 80% of these patients have LAGC. However, information regarding elderly patients with LAGC is very limited. From a pathogenesis point of view, advanced gastric cancer and early gastric cancer have very different biological and molecular characteristics [18]. At the same time, in the predictive nomogram, it was also found that the survival of elderly patients with LAGC might be more strongly influenced by tumour and surgery-related treatments compared with elderly patients with gastric cancer [26]. In this clinical context, the establishment of prognostic nomogram models for predicting the survival of elderly patients with LAGC would be of high clinical application value.

To date, the tumor node metastasis (TNM) staging system is considered to be the ‘gold standard’ for prognostication in oncology [27]. This systematic staging indicates that solid tumours spread sequentially, first from the primary site to the lymphatic system and then to distant organs. Patients are hence classified by both anatomical spread of disease and survival. In the current study, it was also further demonstrated that the TNM staging system, especially lymph node metastasis, remains an important factor for predicting the prognosis of elderly patients with LAGC. However, the TNM system has limitations due to requiring a correlation between anatomical disease progression and increasing stage progression [28]. Patients with equivalent anatomical spread yet different clinical outcomes (recurrence or survival) are forced into the same stage. Indeed, gastric cancer is a highly heterogeneous malignant tumour, and elderly patients with multiple comorbidities and low physiological reserves have distinct biological properties [29]. With the rapid development of molecular genetics, several studies have found that the prognoses of gastric cancer patients with the same TNM stage are not exactly the same or may even differ greatly [30, 31]. Therefore, prognosis prediction for individual elderly patients with LAGC cannot be precisely determined by TNM stage grouping alone and should include other clinical prognostic markers, such as clinical, pathological, and surgical therapy-associated factors, to better direct treatment choice and improve long-term outcomes.

In this study, after using multivariable Cox regression analysis and statistical modelling, it was found that the TNM staging system, demographic variables (age and race), clinicopathological variables (tumour size and differentiation) and surgery-related factors (gastrectomy type, LNR and chemoradiotherapy therapy) were independent risk factors for OS and overall CSS in elderly patients with LAGC. Older age and Indian ethnic backgrounds were associated with poor OS and CSS. The unfavourable prognosis of elderly patients may be explained by poor surgical tolerance and the inability to complete perioperative chemotherapy. Our study also found that Asian-American and Asian populations have the best prognosis for gastric cancer, which was consistent with the reports of some previous studies [26, 32]. The variability in survival rates observed from the external validation set also strongly demonstrated this view. Chemotherapy has long been the standard treatment for advanced gastric cancer [33]. Multiple RCT studies have demonstrated that the combination of radical surgery and perioperative chemotherapy could improve the survival and the quality of life of patients with LAGC [34, 35]. Although the toxicities of classical chemotherapy treatments in older patients are higher and have a greater risk for complications due to the unique and complex pathophysiology, we still found that the prognosis of elderly patients who received chemotherapy was better than that of patients who did not receive chemotherapy.

The degree of differentiation of the tumour is an important index for assessing prognosis and malignant potential, reflecting the biological behaviour of the tumour itself. Even in some tumours, tumour differentiation as an important biologic factor has been incorporated into the staging system [36]. Some studies have reported that the LNR may be superior to the traditional pN stage in predicting tumour patient prognosis [37]. Our data also showed that a high LNR (over 33%) was closely correlated with adverse parameters. In addition, we found that the prognosis of elderly patients with LAGC gradually deteriorated with increasing tumour diameter, which further validated the clinical perception. This is most likely because a larger tumour size might lead to a heavier tumour burden and a greater likelihood of infiltrative growth [38]. In fact, in some solid tumours, T staging is based on the size of the tumour due to its influence on the outcome of our patients. Recent studies have shown that proximal gastric cancer has different clinicopathological features than distal gastric cancer, such as being more prone to deeper gastric wall invasion, lymph node metastasis and postoperative recurrence and having a significantly worse prognosis [39]. We have demonstrated these same findings in our study.

After verifying the discrimination and calibration of the nomograms, it is necessary to further estimate the clinical usefulness of the model. This research introduced decision curve analysis (DCA) to evaluate the clinical usefulness of the nomogram on the basis of the net benefit (defined as the proportion of true positives minus the proportion of false-positives, weighted by the relative harm of a false-positive and false-negative result). DCA was developed as a method to determine whether the use of a prediction model in the clinic to determine if decision-making would do more good than harm. At the same time, unlike traditional biostatistical methods, which only evaluate the accuracy of a model, DCA could tell us whether using a model to aid clinical decision-making would improve outcomes for our patients. By evaluating the net benefit of nomogram-assisted decisions at different threshold probabilities, decision curve analysis visually indicated that the nomogram conferred a high clinical net benefit and might better guide clinical decision-making.

In this study, we found that the factors affecting patient OS and CSS were consistent, reflecting that advanced gastric cancer has an important impact on the survival of elderly patients. Identifying and evaluating risk factors with substantial predictive prognostic value is of great clinical importance in elderly patients with LAGC. Moreover, we can also see that the patients in the external validation set have significantly better CSS and OS than the patients from the SEER database. Apart from racial and age differences, another reason may be that all patients received standardized perioperative care based on the standardized Enhanced Recovery After Surgery (ERAS) protocol among the three externally verified centres. Over the past two years, ERAS has developed appreciably and has become an important part of the perioperative treatment of gastric cancer [40]. Our preliminary study showed that implementation of ERAS principles has the potential to reduce surgical stress, reduce the incidence of postoperative adverse events and improve patient long-term prognosis, particularly for elderly patients [41, 42]. It is necessary to mention that our research has some limitations. First, in elderly patients, some important prognostic information could not be obtained from the SEER database, such as comorbidities, nutrition score, and cardiopulmonary function. Second, with the development of molecular technologies, tumour markers and genetic and molecular heterogeneity of advanced gastric cancer are emerging as important factors influencing prognosis. Such data are missing from the SEER database. Finally, our research is a retrospective study, so a prospective and multicenter study will be necessary in the future to further validate the results.

## Conclusion

The present study is the first to establish a nomogram model of CSS and OS based on a large population database for elderly patients with LAGC to effectively predict the survival rate. The prediction model showed good discrimination and calibration through internal and external validation. As a valid clinical tool, the nomogram achieves personalized precision prediction of patient survival for elderly patients with LAGC and improves the clinical decision-making power of clinicians.

## Data Availability

Original data are available from the corresponding author, Yanbing Zhou, on request.
